# Hidden hazard of remote cerebellar hemorrhage after cervical spine surgery: Case report

**DOI:** 10.1016/j.ijscr.2025.111571

**Published:** 2025-06-26

**Authors:** Barnabas Obeng-Gyasi, Emma Stephens Love, Mugtaba Swar-Eldahab, Matthew Blackwell, Anoop S. Chinthala, Gordon Mao

**Affiliations:** Indiana University School of Medicine, Department of Neurological Surgery, 355 W 15th St, Suite 5100, Indianapolis, IN 46202, USA

**Keywords:** Remote cerebellar hemorrhage, Spinal surgery complications, Cerebrospinal fluid leak, Dural tear, Postoperative monitoring, Case report

## Abstract

**Introduction and importance:**

Remote cerebellar hemorrhage (RCH) is a rare but potentially serious complication of spinal surgery, particularly after decompressive procedures and instrumented fusion. The complex interplay between cerebrospinal fluid leaks and hemorrhage risk necessitates a high index of suspicion and prompt management to optimize patient outcomes.

**Case presentation:**

We present a case of RCH in a 50-year-old man with hypertension, non-insulin dependent diabetes mellitus, and cervical myeloradiculopathy following a C3-C6 laminectomy with posterior instrumented fusion. Despite an initially benign presentation, the patient developed severe positional headaches, nausea, vomiting, and lethargy. A head CT on postoperative day five revealed a right cerebellar hemispheric hemorrhage with obstructive hydrocephalus. Intensive care management, including strict blood pressure control, serial neuroimaging, and close neurological monitoring, led to hemorrhage stabilization and discharge on postoperative day 18.

**Clinical discussion:**

RCH is believed to result from venous infarction secondary to rapid cerebrospinal fluid loss, often associated with dural tears. Even minor cerebrospinal fluid leaks can precipitate significant complications, underscoring the importance of early recognition and tailored management. Patient positioning and subfascial drainage strategies during surgery should be optimized to mitigate risks, and vigilant postoperative monitoring is critical for timely intervention.

**Conclusion:**

This case underscores the importance of meticulous surgical technique, vigilant postoperative care, and the judicious use of imaging in managing RCH. It also highlights that radiographic severity does not always dictate the need for aggressive surgical intervention and emphasizes the significance of recognizing postoperative headaches as a potential sign of intracranial bleeding.

## Highlights

• Remote cerebellar hemorrhage (RCH) can occur after spinal surgery, even with minor CSF leaks.
• Early imaging and strict blood pressure control are essential for RCH management.
• Meticulous dural repair and drain management help minimize CSF loss and RCH risk.
• Postoperative surveillance is crucial for timely detection and intervention in RCH.

## 1 Introduction

Remote cerebellar hemorrhage (RCH) is a rare but significant complication of spinal surgery, with an incidence of 0.0657 % [1]. It is most frequently associated with decompressive procedures and instrumented fusion for spinal canal stenosis but has also been observed after spinal tumor debulking and disc herniation surgeries [[1], [2], [3]]. Characterized by the ‘zebra sign’ bleeding pattern resulting from venous infarction following rapid cerebrospinal fluid (CSF) loss, RCH is often linked to intraoperative dural lesions, which are present in up to 93 % of cases [3,4]. Clinically, RCH presents with a spectrum of symptoms, from benign headaches and nausea to severe neurological impairments such as altered mental status and cerebellar dysfunction [1,[5], [6], [7]]. The risk factors for RCH include arterial hypertension, extensive CSF loss, and postoperative subfascial drains, necessitating meticulous surgical technique and vigilant perioperative care [1,8].

This case is notable for the development of RCH following a C3-C6 laminectomy and posterior instrumented fusion complicated by a minor durotomy. Managed at an academic tertiary care center, the patient benefited from advanced critical care and interdisciplinary expertise, culminating in a favorable outcome. This report highlights the importance of recognizing RCH as a potential postoperative complication and emphasizes the need for early detection and tailored management strategies.

This case report has been prepared and reported in accordance with the SCARE 2025 guidelines for surgical case reports [9].

## 2 Presentation of case

A 50-year-old left-handed white male, employed as a general manager at a restaurant, initially presented for evaluation of worsening chronic neck pain and progressive left arm weakness and numbness radiating into his thumb, index, and middle fingers over the past year. Symptoms disrupted his sleep and interfered significantly with his work. His medical history included hypertension, non-insulin-dependent diabetes mellitus, hyperlipidemia, obstructive sleep apnea, and chronic neck pain. He had been diagnosed with mild cervical myelopathy seven years prior but was advised against surgery due to concerns over adjacent-level disease.

On examination prior to surgery, he had diminished strength in his left hand (4/5 grasp) and biceps (4+/5), but otherwise no focal neurological deficits. MRI imaging demonstrated severe multilevel cervical spinal stenosis with congenital and degenerative changes.

He underwent a C3–C6 laminectomy and posterior instrumented fusion under general anesthesia in the prone position. A small intraoperative dural tear at C3–C4 occurred, resulting in minimal cerebrospinal fluid (CSF) leakage, and was repaired with Tachosil fibrin patch and Vistoseal fibrin glue. A subfascial 10Fr Hemovac drain was placed, which initially produced moderate output (150 cc on postoperative day [POD]1, 190 cc on POD2).

**Fig. 1:**
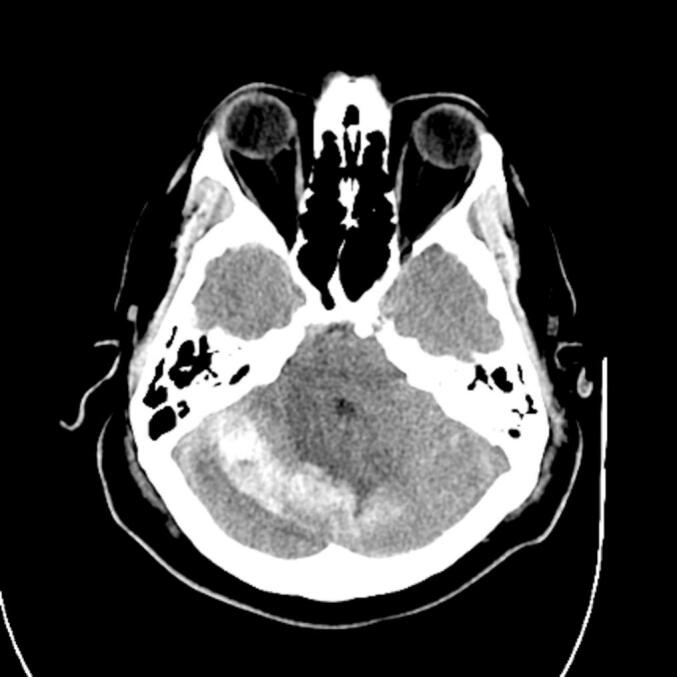
Head CT without IV contrast POD5 demonstrating a 1.7 cm × 4.9 cm × 1.5 cm right cerebellar hemorrhage with mild mass effect on the fourth ventricle.

Immediately postoperatively, he exhibited full motor strength and intact sensation but reported right thumb pain. By POD2, he developed severe positional headaches with nausea and vomiting. On POD3, the drain output decreased significantly (30 cc), and he experienced difficulty urinating, requiring intermittent catheterization. Despite minimal drain output on POD4, his severe headaches persisted. Throughout this period, there were no cerebellar signs such as dysmetria, ataxia, or nystagmus on examination. On POD5, he developed lethargy and fever (38.5 °C), prompting a head CT, which revealed a right cerebellar hemispheric hemorrhage measuring 1.7 cm × 4.9 cm × 1.5 cm with associated obstructive hydrocephalus, corresponding to an intracerebral hemorrhage (ICH) score of 1 (Fig. 1). Imaging was initially deferred given the absence of focal neurological deficits and a stable neurological exam, but was obtained urgently on POD5 after the onset of lethargy and fever. The Hemovac drain was removed at this time.

### 2.1 Treatment

Given the obstructive hydrocephalus and 4.9 cm hemorrhage, the patient was transferred to the intensive care unit (ICU) for close monitoring. He was maintained in a head-up position (30 degrees) with strict systolic blood pressure control below 140 mmHg using intravenous medications and administered 3 % hypertonic saline as needed for cerebral edema.

**Fig. 2:**
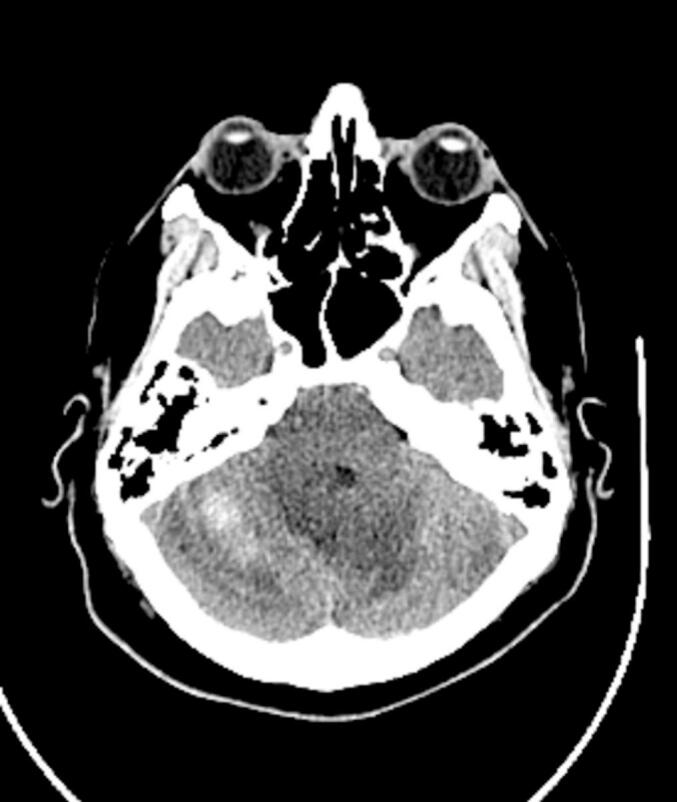
Head CT without IV contrast POD15 demonstrating improvement of right cerebellar hemorrhage during inpatient course.

**Fig. 3:**
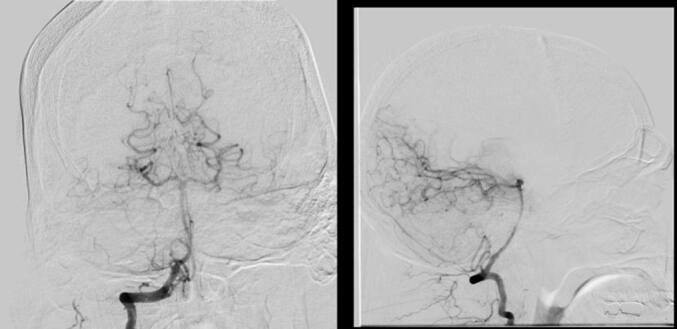
6-vessel digital subtraction angiography ruling out the presence of an aneurysm or arteriovenous malformation.

Over 11 days in the ICU, serial head CT scans showed gradual improvement in both the hemorrhage and hydrocephalus (Fig. 2). A 6-vessel digital subtraction angiography ruled out aneurysms, dural venous sinus thrombosis, and arteriovenous malformations (AVMs) as potential contributors (Fig. 3). Given the stable neurological exam and rapid clinical improvement, no further surgical intervention was pursued. Although the hemorrhage measured nearly 5 cm with obstructive hydrocephalus, the patient's stable neurological exam, preserved consciousness, and rapid clinical improvement supported conservative management. Surgical intervention such as ventriculostomy or hematoma evacuation was deemed unnecessary given the resolving radiographic and clinical course.

### 2.2 Outcome and follow-up

Following the removal of the Hemovac drain on POD5, the patient experienced gradual headache relief and improved mental status. Serial head CTs showed no progression and eventual stabilization. He was discharged on POD18 with a head CT showing resolving hemorrhage, edema, and ventriculomegaly.

**Fig. 4:**
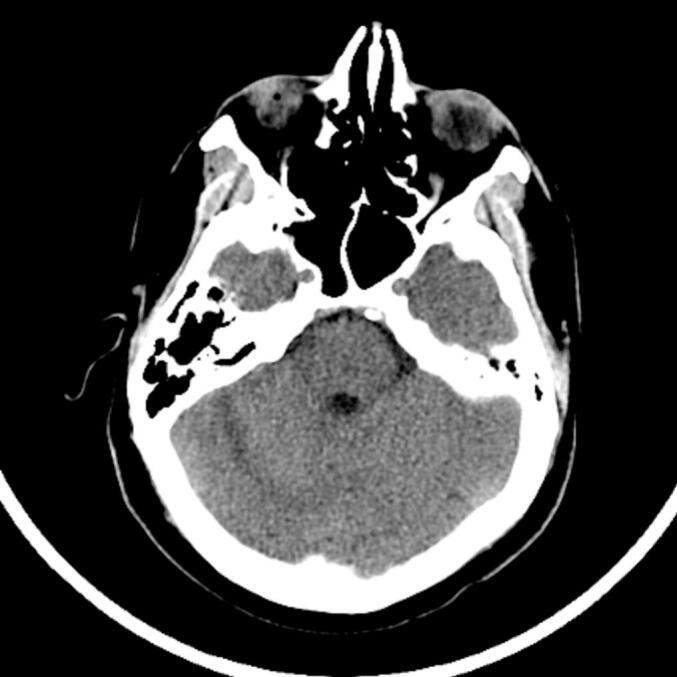
Head CT without IV contrast 1 month postoperatively demonstrating complete resolution of cerebellar hemorrhage.

At follow-up 26 days after the initial hemorrhage was identified (POD31), he demonstrated good mobility with minor balance issues and no headache. A repeat CT scan at the time confirmed complete resolution of the hematoma and ventriculomegaly (Fig. 4). He resumed work three months after surgery.

## 3 Discussion

RCH following spinal surgery is rare but clinically significant, with over 60 reported cases. Initially described by Chadduck in 1981 [10], it is most often attributed to venous hemorrhagic infarction due to cerebellar sagging from rapid CSF loss after dural tears [[1], [2], [3]]. These findings highlight the importance of meticulous surgical technique to minimize dural injury and reduce RCH risk [1].

In our case, a minor intraoperative dural tear was initially evaluated to be low risk in the immediate post-operative period, yet RCH developed despite the small CSF leak. Notably, RCH risk does not scale proportionally with CSF loss; even minor leaks require vigilant postoperative monitoring [1,2,11]. This challenges traditional assumptions and underscores the necessity of close surveillance regardless of leak size.

Although a prior systematic review and meta-analysis found no clear association between surgical location and hemorrhage risk [2], the proximity of the CSF leak to the posterior fossa in our case may have played a significant role. RCH frequently follows lumbar decompression with instrumented fusion due to the prevalence of degenerative lumbar disease and dural injuries during pedicle screw insertion [1,12]. These occult dural tears may be more prone to RCH than intentional openings sealed intraoperatively [1,3,12].

Compared to RCH, supratentorial ICH after spinal surgery is less common and generally involves distinct mechanisms, such as hypertension or coagulopathy [1,13]. In contrast, infratentorial hemorrhages, including RCH, typically arise from venous infarctions linked to cerebellar sagging and altered venous drainage caused by CSF loss [13].

Patient positioning influences the pressure gradient across the cranium and spinal canal, affecting CSF dynamics and hemorrhage risk [1,14,15]. Proper patient positioning and judicious use of subfascial drains can limit CSF loss without inducing harmful low-pressure states [1,8]. Postoperative headaches, even if seemingly benign, warrant immediate evaluation as they may signal intracranial bleeding [1,4,15].

Evidence supports the use of subfascial drains to aid in wound healing and prevent cerebrospinal fluid (CSF) fistulas. The proposed mechanism involves reducing subfascial pressure buildup and tension on the surgical wound [[16], [17], [18]]. Once the tissue has time to strengthen, the drain can be removed. The backpressure created by the fascia and any potential pseudomeningocele formation is thought to reach an equilibrium with the subdural CSF, stopping additional flow. This mechanism allows the dural flaps to approximate and adhere, promoting effective closure of the dural defect. While the duration of the drain varies, studies have demonstrated a significant reduction in reoperation rates for persistent CSF leaks when subfascial drains are used for 5 to 7 days postoperatively compared to no drainage [19]. The use of subfascial drains has been associated with fewer reoperations, reduced need for inpatient rehabilitation, and lower readmission rates, thereby promoting better overall outcomes in spinal surgery patients. Other CSF diversion strategies such as lumbar drainage or re-exploration were not pursued given the minimal leak, effective primary repair, and spontaneous clinical improvement following drain removal.

RCH presentations vary, and our patient's early lethargy and fever diverged from the more common altered consciousness or focal deficits [3,12]. While most RCH cases manifest clinically within the first 72 h—42.1 % within 24 h and another 40.3 % by 72 h—later presentations still occur, underscoring the need for sustained vigilance and timely imaging even several days post-surgery [3].

The patient had hypertension, a less common risk factor for RCH in spine surgeries compared to its higher prevalence in RCH after supratentorial craniotomies [3,12]. The elective nature of his surgery might have influenced the management of anticoagulant or antiplatelet therapies. Notably, his imaging showed a pure intracerebellar hemorrhage pattern, more associated with spinal surgery RCH than with supratentorial procedures [3,12]. Despite this, he did not require further surgical intervention and achieved a favorable outcome, aligning with the experiences of approximately three-quarters of patients in similar situations [1,3,12]. The diverse clinical presentations and outcomes associated with RCH in spinal surgeries, as demonstrated by our patient's case, highlight the need for prompt diagnosis and tailored management strategies.

Imaging findings play a pivotal role in guiding management decisions for RCH. After cervical spinal surgery, RCH often resolves spontaneously, and limited hemorrhages typically respond well to conservative management. Close monitoring and follow-up CT imaging are essential [15]. Notably, concerning radiological features do not necessarily mandate immediate surgical intervention, particularly if the hemorrhage is venous in origin [1,2,6,7].

In patients with arterial hypertension or following dural tears, the use of subfascial suction drainage can increase the risk of intracranial hemorrhage (IH). Prompt recognition of neurological decline after spinal surgery through cranial imaging is therefore critical, especially in revision surgeries, to prevent adverse outcomes [1,2]. In our patient's case, we ensured diligent monitoring and maintained systolic blood pressure using a nicardipine drip to mitigate cerebral edema. Recovery is influenced by a treatment approach tailored to the severity and location of the IH.

When managing post-spinal surgery hemorrhage, it is critical to exclude other etiologies for intracranial bleeding. Cerebral angiography can effectively rule out vascular malformations such as AVMs, aneurysms, or dural venous sinus thrombosis. Identifying these conditions is essential, as they may require more aggressive interventions [20]. A comprehensive evaluation that balances clinical presentation and imaging findings is fundamental to guiding appropriate treatment for RCH.

## 4 Conclusion

Vigilant monitoring of even minor CSF leaks following spinal surgery is crucial, as they can precipitate complications like RCH. This case underscores the non-linear relationship between CSF loss and hemorrhage risk, emphasizing rigorous surveillance and tailored management. Meticulous patient positioning, careful dural repair, and proactive postoperative care help mitigate RCH risk and highlight the need for sustained diligence.

## Submission statement

This manuscript is original and has not been submitted elsewhere in part or in whole.

## Consent

Written informed consent was obtained from the patient for publication of this case report and accompanying images. A copy of the written consent is available for review by the Editor-in-Chief of this journal on request.

## Ethical approval

According to the guidelines of the Indiana University Human Research Protection Program (HRPP), this single patient case study does not constitute human subjects research and is exempt from Institutional Review Board (IRB) review, as it does not involve identifiable private information.

## Funding

This research did not receive any specific grant from funding agencies in the public, commercial, or not-for-profit sectors.

## Author contribution

Barnabas Obeng-Gyasi – Conceptualization; Investigation; Project administration; Supervision; Roles/Writing - original draft; and Writing - review & editing.

Emma Stephens Love – Conceptualization; Investigation; Roles/Writing - original draft; and Writing - review & editing.

Mugtaba Swar-Eldahab – Conceptualization; Investigation; Roles/Writing - original draft; and Writing - review & editing.

Matthew Blackwell – Investigation; Roles/Writing - original draft; and Writing - review & editing.

Gordon Mao – Conceptualization; Project administration; Supervision; Roles/Writing - original draft; and Writing - review & editing.

## Guarantor

Barnabas Obeng-Gyasi.

Emma Stephens Love.

Mugtaba Swar-Eldahab.

Matthew Blackwell.

Anoop S. Chinthala.

Gordon Mao.

## Research registration number

None.

## Conflict of interest statement

There are no conflicts of interest to disclose.
